# Exome sequencing reveals recurrent *REV3L* mutations in cisplatin-resistant squamous cell carcinoma of head and neck

**DOI:** 10.1038/srep19552

**Published:** 2016-01-21

**Authors:** Kie Kyon Huang, Kang Won Jang, Sangwoo Kim, Han Sang Kim, Sung-Moo Kim, Hyeong Ju Kwon, Hye Ryun Kim, Hwan Jung Yun, Myung Ju Ahn, Keon Uk Park, Kalpana Ramnarayanan, John R. McPherson, Shenli Zhang, Je-Keun Rhee, André L. Vettore, Kakoli Das, Takatsugu Ishimoto, Joo Hang Kim, Yoon Woo Koh, Se Hun Kim, Eun Chang Choi, Bin Tean Teh, Steven G. Rozen, Tae-Min Kim, Patrick Tan, Byoung Chul Cho

**Affiliations:** 1 Cancer Science Institute of Singapore, National University of Singapore, Center for Translational Medicine, Singapore, Singapore; 2 Program in Cancer and Stem Cell Biology, Duke-NUS Graduate Medical School, Singapore, Singapore; 3 JE-UK Institute for Cancer Research, JEUK Co., Ltd., Gumi-City, Kyungbuk, Korea; 4 Severance Biomedical Science Institute, Yonsei University College of Medicine, Seoul, Korea; 5 Division of Medical Oncology, Department of Internal Medicine, Yonsei Cancer Center, Seoul, Korea; 6 Department of Pharmacology, Pharmacogenomic Research Center for Membrane Transporters, Brain Korea 21 PLUS Project for Medical Science, Seoul, Korea; 7 Department of Pathology, Yonsei University College of Medicine, Seoul, Korea; 8 Department of Hematology-Oncology, Chungnam National University, Daejeon, Korea; 9 Division of Hematology-Oncology, Department of Medicine, Samsung Medical Center, Sungkyunkwan University School of Medicine, Seoul, Korea; 10 Department of Hematology-Oncology, Keimyung University, Daegu, Korea; 11 Duke-NUS Centre for Computational Biology, Duke-NUS Graduate Medical School, Singapore, Singapore; 12 Department of Medical Informatics, College of Medicine, The Catholic University of Korea, Seoul, Korea; 13 Department of Otorhinolaryngology, Yonsei University College of Medicine, Seoul, Korea; 14 Laboratory of Cancer Epigenome, National Cancer Centre Singapore, Singapore, Singapore; 15 Genome Institute of Singapore, Singapore, Singapore

## Abstract

Dacomitinib, an irreversible pan-HER inhibitor, had shown modest clinical activity in squamous cell carcinoma of head and neck (SCCHN) patients. Therefore, validated predictive biomarkers are required to identify patients most likely to benefit from this therapeutic option. To characterize the genetic landscape of cisplatin-treated SCCHN genomes and identify potential predictive biomarkers for dacomitinib sensitivity, we performed whole exome sequencing on 18 cisplatin-resistant metastatic SCCHN tumors and their matched germline DNA. Platinum-based chemotherapy elevated the mutation rates of SCCHN compared to chemotherapy-naïve SCCHNs. Cisplatin-treated SCCHN genomes uniquely exhibited a novel mutational signature characterized by C:G to A:T transversions at CCR sequence contexts that may have arisen due to error-prone translesional synthesis. Somatic mutations in *REV3L*, the gene encoding the catalytic subunit of DNA polymerase ζ involved in translesional synthesis, are significantly enriched in a subset of patients who derived extended clinical benefit to dacomitinib (*P* = 0.04). Functional assays showed that loss-of-function of REV3L dramatically enhanced the sensitivity of SCCHN cells to dacomitinib by the loss of both translesion synthesis and homologous recombination pathways. Our data suggest that the ‘platinum’ mutational signature and inactivation of REV3L may inform treatment options in patients of recurrent SCCHN.

Squamous cell carcinoma of the head and neck (SCCHN) is the seventh most common cancer worldwide^1^. Recurrent or metastatic diseases will occur in 50–60% of patients. Although cisplatin-based chemotherapy remains the backbone of palliative treatment in recurrent or metastatic SCCHN, most patients die within one year after the recurrence.

Epidermal growth factor receptor (EGFR/HER1), one of the four HER family receptors (EGFR/HER1, HER2, HER3, HER4), is an attractive molecular target in SCCHN. EGFR is almost universally expressed in SCCHN and its overexpression is correlated with poor prognosis^2^. In this context, EGFR-targeted agents have been extensively studied in SCCHN and these include cetuximab (an anti-EGFR antibody), gefitinib/erlotinib (selective EGFR inhibitors), and afatinib/dacomitinib (irreversible pan-HER inhibitors). However, the clinical benefit of these targeted agents is typically modest with reported response rate of 1.4%–20.8% and progression-free survival of 1.8–3.9 months^3^,^4^,^5^,^6^,^7^. Therefore, there is a pressing need to identify molecular predictors to evaluate the efficacy of EGFR inhibitors in order to maximize the clinical benefit of EGFR inhibitors.

Cancer genome sequencing has revealed that specific mutagens or mutagenic processes produce characteristic patterns of somatic mutations, called mutation signatures, in the cancer genome^8^,^9^. Cisplatin is highly mutagenic in experimental models and its cytotoxicity is mainly mediated through the formation of DNA adducts. DNA damage and the resulting mutations, if not repaired by DNA repair mechanisms, accumulate during cisplatin-based chemotherapy and may have impact on tumor biology and drug resistance. In this regard, deciphering the cisplatin-induced mutation signature by whole-exome sequencing (WES) of cisplatin-resistant tumors may provide valuable information on novel therapeutic targets, which have not previously been identified from WES studies of chemotherapy-naïve SCCHN. This information is particularly important for recurrent and/or metastatic SCCHN, because EGFR-targeted agents have often been tested in platinum-resistant metastatic SCCHN.

## Results and Discussion

### Patient Characteristics and Study Design

**Figure 1:**
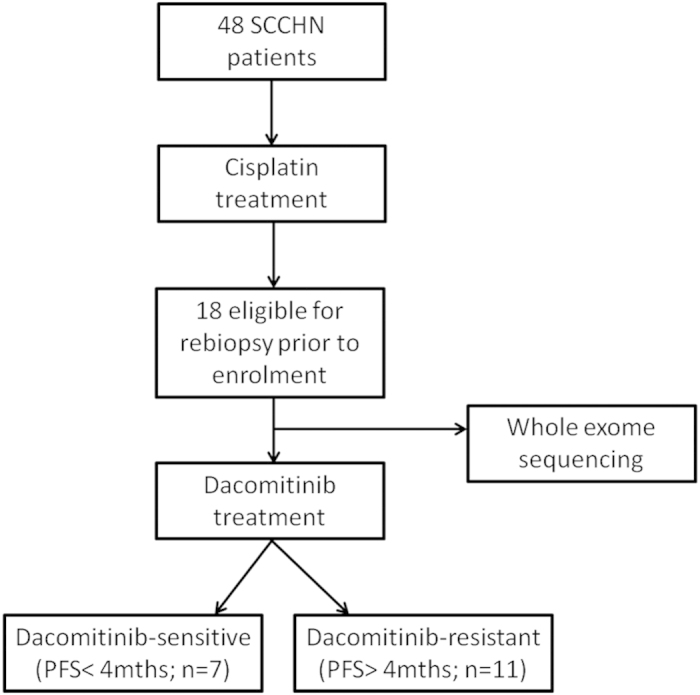
Study design and classification of dacomitinib-sensitive and –resistant patients. SCCHN patients were enrolled into a phase II clinical trial exploring the efficacy of dacomitinib in recurrent SCCHN.

Of 48 patients enrolled in the trial, 18 patients had received cisplatin-based chemotherapy and were eligible for rebiopsy, based on presence of accessible tumor lesions and minimal risk of complications in multidisciplinary team discussion (Fig. 1). The tumor characteristics and the response to dacomitinib were described (Supplementary Table S1 and Supplementary Table S2).

Based on response to dacomitinib, we classified tumors into dacomitinib-sensitive (*n* = 7) or dacomitinib-resistant (*n* = 11) tumors. Dacomitinib-sensitive tumors were defined as progression-free survival (PFS) ≥4 months on dacomitinib according to Response Evaluation Criteria in Solid Tumors (RECIST 1.1), because most salvage therapies with either cytotoxics or EGFR inhibitors in platinum-resistant SCCHN have shown PFS of approximately 2 months^3^,^4^,^5^,^10^,^11^.

### Mutational Landscape of Recurrent SCCHN Tumors

The WES results from germline and tumor pairs were summarized (Supplementary Table S3). Tumor-germline pairs were sequenced to a mean coverage of 153X and 91X. Overall, we identified 10,115 somatic coding single nucleotide variants (SNVs) and small insertions/deletions (indels). The full list of somatic mutations is provided (Supplementary Table S4). Mutation rates were highly variable across tumors (4.8 to 80.9 mutations/Mb; median of 13.7 mutations/Mb).

**Figure 2:**
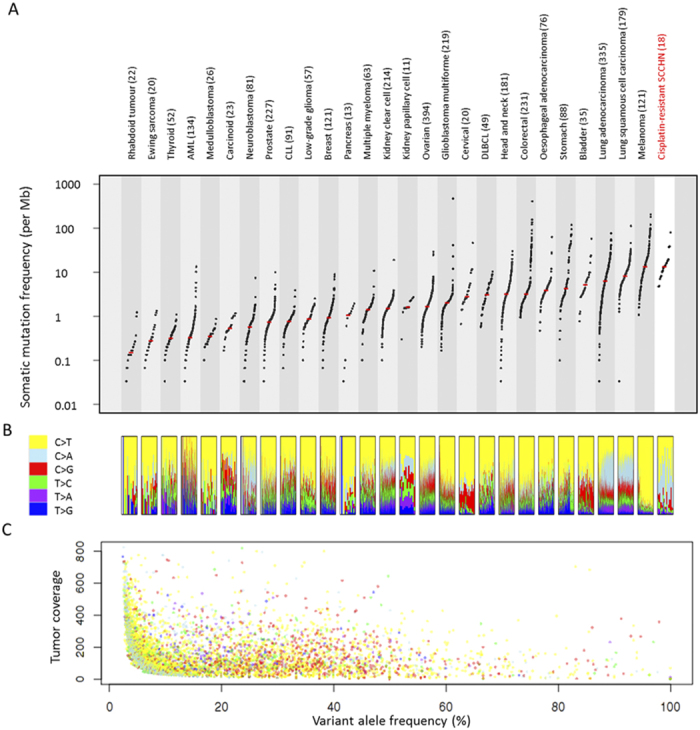
Mutational landscape of cisplatin-resistant SCCHN. (**A**) Mutation rate in cisplatin-resistant SCCHN compared to mutation rate in other major tumor types. Each dot represents the mutation rate in a single tumor. The median mutation rate is indicated by red bar (**B**) Relative proportion of the six mutational spectra as indicated in the legend box in each tumor sample. (**C**) Variant allele frequency in SCCHN. Each dot represents the variant allele frequency of a predicted mutation. Colors indicate the mutational spectra as indicated by legend box in Fig. 2B. Design and data for Fig. 5A,B are obtained from previous literature^13^.

Compared with the 279 SCCHN mutation profiles from chemotherapy-naïve SCCHN of The Cancer Genome Atlas consortium (TCGA)^12^, the 18 SCCHN cisplatin-treated samples exhibited significantly higher mutation rates (median 13.7 *vs* 4.4 mutations/Mb; Wilcoxon test, *P*=6.8 × 10^–8^). The mutation rate in our cohort of cisplatin-treated SCCHN is comparable to or higher than the mutation rates observed in melanoma (median 13.2 mutations/Mb), lung squamous cell carcinoma (median 8.2 mutation/Mb) and lung adenocarcinoma (median 6.4 mutation/Mb), the cancer types associated with chronic exposure to mutagenic agents (Fig. 2A)^13^.

We observe a variable Ti/Tv (transition-vs-transversion) ratios across the genomes (range 0.19–3.93; median 0.76), indicating distinct mutational processes in different patients (Fig. 2B). The relatively low Ti/Tv ratios of the 18 SCCHN genomes are largely attributed to the overrepresentation of C > A transversions in some patients. Most predicted somatic mutations in our cohort have low variant allele frequency, despite tumor purity estimate of more than 70%. The majority (76.1%) of the predicted SNVs are found in less than 20% of all the tumor sequencing reads, including most (91.9%) of the cisplatin-associated C > A transversions (Fig. 2C).

### Mutational Signature in Recurrent SCCHN

**Figure 3:**
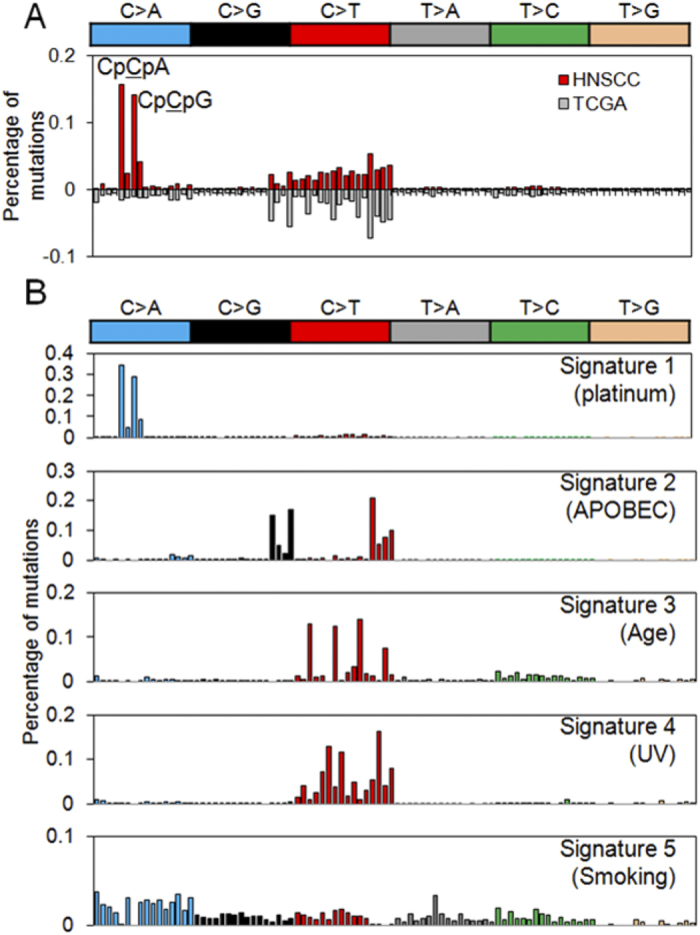
Mutational signatures in recurrent SCCHN. (**A**) Relative frequencies of 96 trinucleotides are shown for cisplatin-treated and cisplatin-naïve SCCHN above and below, respectively. (**B**) The deciphered five mutational signatures are shown for their relative frequencies across 96 trinucleotides. Note that signature 1 (platinum) contributes to a substantial level of difference between cisplatin-treat and -naive SCCHN genomes in (**A**).

We extracted five distinct mutational signatures representing unique mutational processes operative in SCCHN genomes (Fig. 3A and Supplementary Figure S1). In addition to the four signatures that have been previously reported to be associated with SCCHN^8^ (i.e., APOBEC, age, ultraviolet, and smoking corresponding to signatures 2 – 5; Fig. 3B), we identified a mutational signature that has not been previously reported in a large-scale analysis across 30 tumor types (signature 1; Fig. 3B)^8^. This signature is characterized by the prominence of C > A mutations mainly occurring on CpCpA and CpCpG trinucleotide contexts.

The sequence composition of this novel signature is associated with the mechanistic action of cisplatin. Platinum preferably forms intrastrand crosslinks between purines with the Pt-GpG adducts being the most frequently formed DNA adducts^14^. In addition, the substantial C >A mutation on CpC dinucleotides, especially those preceding purines, suggested the preferential misincorporation of adenine opposite the 5′G of the Pt-GpG adducts. This preference for insertion of adenine across DNA lesion is reminiscent of the “A-rule” phenomenon observed in cells undergoing translesion DNA synthesis (TLS)^15^. We therefore, proposed that the major substitution associated with cisplatin are TpGpG > TpTpG and CpGpG > CpTpG and this sequence preference may arise from activated error-prone translesion DNA replication activity on the DNA binding sites of cisplatin^16^. Thus, signature 1 in our dataset had been annotated as ‘platinum’. This mutational signature was unlikely to be associated with prior exposure to radiation, because a significant fraction of DNA damage produced by ionizing radiation comes from free radicals generated during the radiolysis of water, leading to single-strand breaks of DNA^17^.

### REV3L is Recurrently Mutated in Dacomitinib-sensitive Tumors

To identify significantly mutated genes in recurrent SCCHN genomes, we performed the mutational significance test using the Genome MuSiC algorithm^18^. We include only variants with allele frequency of more than 20% in this analysis, reasoning that low frequency variants not subjected to stringent selection are likely to be passenger mutations from the diverse mutational processes or subclonal events.

**Figure 4:**
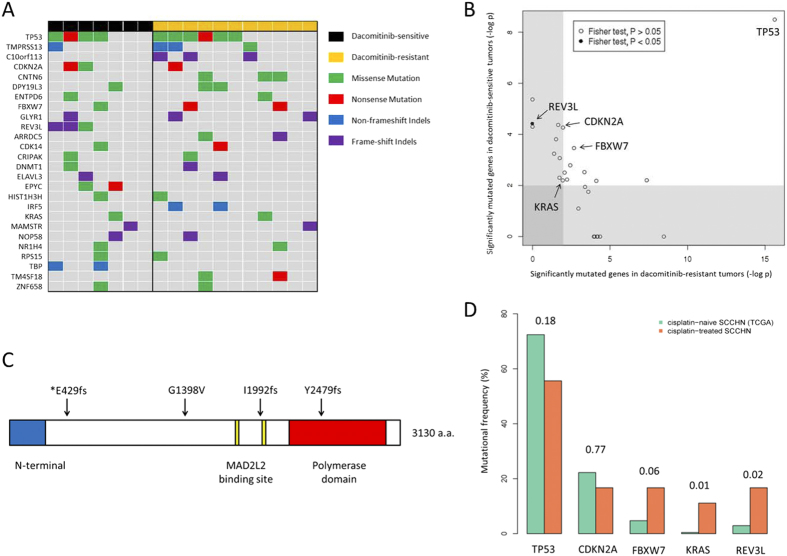
REV3L mutation in dacomitinib-sensitive SCCHN. (**A**) Recurrent mutations in metastatic SCCHN. All recurrently mutated genes that are significantly mutated in either dacomitinib-sensitive patients, dacomitinib-resistant patients or the overall cohort are included in this figure. (**B**) Putative biomarkers for dacomitinib responsiveness. Figure showed the mutational significance (- log uncorrected *P* value from genome MuSiC’s convolution test) in dacomitinib-sensitive and dacomitinib-resistant patients. Gene with significant enrichment in dacomitinib-sensitive compared to dacomitinib-resistant cohort are indicated (Fisher exact test *P* < 0.05). (**C**) Mapping of somatic mutations in *REV3L*. The single patient with low frequency *REV3L* mutation is indicated with asterisk. (**D**) Mutational frequency of *TP53*, *CDKN2A*, *FBXW7*, *KRAS* and *REV3L* in cisplatin-treated SCCHN (*n* = 18) and TCGA’s cisplatin-naïve SCCHN (*n* = 279). *P* value (Fisher exact test) between the mutation frequencies for each genes in the two datasets were indicated.

Using this approach and focusing on genes recurrently mutated in more than one patient, we identified 24 mutated genes showing trends towards higher mutation rate compared to the background mutation rate (convolution test, FDR < 0.2; Supplementary Table S5). This set of genes includes well known driver cancer genes including *TP53*, *CDKN2A*, *FBXW7* and *KRAS*. Applying the same procedure on the dacomitinib-sensitive patients (*n* = 7; Supplemetary Table S6) and dacomitinib-resistant patients (*n* = 11; Supplementary Figure S7) identified another two genes (*REV3L* and *EPYC*) to be frequently mutated in dacomitinib-sensitive patient but not in the overall cohort. No genes were found to be significant in dacomitinib-resistant but insignificant in the overall cohort. Along with *REV3L* and *EPYC*, a total of 26 predicted significantly mutated genes are presented in Fig. 4A.

For the 26 somatic variants, only *REV3L* showed significant enrichment to dacomitinib-sensitive tumors (*P* = 0.04; Fisher’s exact test; Fig. 4B). Most of identified *REV3L* mutations are apparently loss-of-functional events (one missense mutation and two frameshifting indels; Fig. 4C), suggesting that the loss of functions in *REV3L* may confer sensitivity to dacomitinib. All three *REV3L* variants were not present in dbSNP135, 1000Genomes or the NIH-NHLBI6500 exome databases, indicating that these mutations may be pathogenic.

We next compared the mutation frequency of *TP53*, *CDKN2A*, *FBXW7*, *KRAS* and *REV3L* in our cohort of cisplatin-resistant SCCHN against TCGA SCCHN exomes. We observed significant enrichment of mutations in *REV3L* (Fisher exact test, *P* = 0.02) and *KRAS* (Fisher exact test, *P* = 0.01) in our dataset compared to TCGA (Fig. 4D). Notably, two cisplatin-resistant patients harboured the hotspot G12D *KRAS* mutation, an alteration that is rarely observed in treatment-naive SCCHN^12^,^19^,^20^. Consistent with this, we observed an additional REV3L frameshift indel in one dacomitinib-sensitive tumor (T15) at a very low frequency (<5%). Strongly supporting our hypothesis, all three *REV3L* mutations were confirmed in cisplatin-treated samples but not in cisplatin-naïve samples using bi-directional Sanger sequencing (Supplementary Figure S2). The final *REV3L* mutations were predicted at below Sanger’s detection level and we are unable to confirm this mutation.

### REV3L Inactivation Confers Sensitivity to Dacomitinib

Inactivation of REV3L induces accumulation of persistent DNA damages containing unrepairable DNA double strand breaks (DSBs) in cancer cells, leading to suppression of tumor cell growth and induction of cellular senescence^21^. EGFR inhibitors induce cell cycle arrest and cellular senescence in tumor cells sustaining DNA DSBs by suppressing DNA repair capacity^22^,^23^. Therefore, we hypothesize that there is a favourable antitumor interaction between REV3L inactivation and dacomitinib via accumulation of unrepairable DNA DSBs.

We first measured the mRNA expression of REV3L in two dacomitinib-sensitive cells (CAL27, CAL33) and two dacomitinib-resistant cells (FADU, MSKQLL2). All four cell lines showed similar levels of REV3L mRNA expressions, regardless of their sensitivity to dacomitinib, suggesting that significant REV3L depletion may be required for meaningful functional effects on dacomitinib sensitivity (Supplementary Figure S3). We selected two cell lines (FADU, MSKQLL2) with strong resistance to dacomitinib (IC50 > 1 μM) for further analysis.

**Figure 5:**
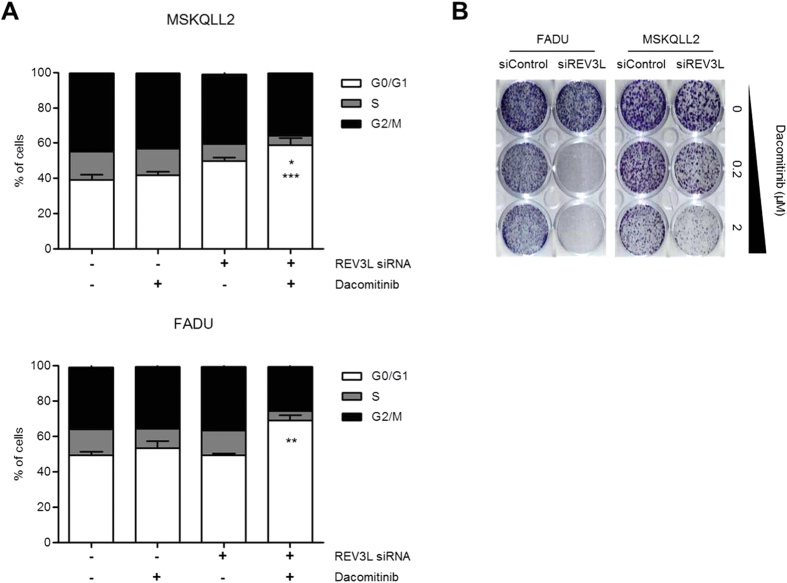
Suppression of REV3L enhances sensitivity to dacomitinib in FADU and MSKQLL2 cells. (**A**) Twenty-four hours after transfection with control siRNA and siREV3L, cell were treated with dacomitinib for 72 h. Cell cycle distribution was measured by propidium iodide staining and subsequent FACS analysis. Error bars indicate mean ± SEM (*n* = 3). *P < 0.05, siREV3L *vs*. combination of siREV3L and dacomitinib; **P < 0.01, siREV3L (or dacomitinib) *vs*. combination of siREV3L and dacomitinib; *** *P* < 0.001, dacomitinib *vs*. combination of siREV3L and dacomitinib. (**B**) Control or siREV3L-transfected FADU and MSKQLL2 cells were treated with dacomitinib at the indicated concentration for 14 days before staining with crystal violet.

Next, we evaluated whether REV3L depletion may enhance sensitivity to dacomitinib in FADU and MSKQLL2 cells. Compared to dacomitinib or siREV3L alone, combined treatment of dacomitinib and siREV3L significantly induced G0/G1 arrest in both cells (Fig. 5A). Consistent with the effects of siREV3L on cell cycle arrest, the antiproliferative effect of dacomitinib in both cells was significantly increased by cotreatment of siREV3L in colony formation assays (Fig. 5B). Furthermore, cotreatment of dacomitinib and siREV3L significantly induced cellular senescence, as evidenced by staining of a senescence biomarker, acidic β-galactosidase (Supplementary Figure S4). In contrast, synergistic antitumor effects were not observed by the combined treatment of siREV3L with paclitaxel or cisplatin, suggesting that loss of REV3L function may contribute to selective sensitization of tumor cells to dacomitinib (Supplementary Figure S5).

Together, these data suggest that REV3L inactivation enhance response to dacomitinib by inducing cell cycle arrest and cellular senescence.

### Cotreatment of siREV3L and Dacomitinib produced Synergistic Antitumor effects by Inhibition of Homologous Recombination repair

**Figure 6:**
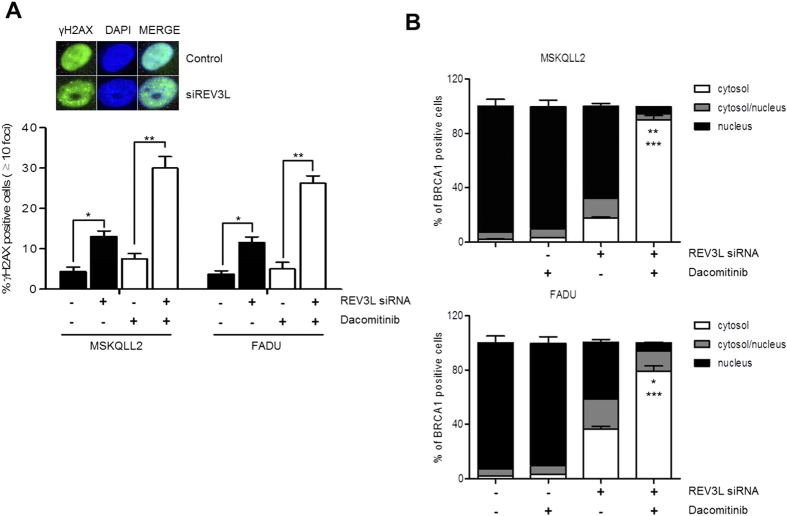
Dacomitinib induces persistent DNA damage in REV3L-depleted head and neck cancer cells. (**A**) Cells were transfected with control siRNA or siREV3L for 24 h, and treated with DMSO or 1 μM Dacomitinib for 72 h. Shown is the percentage of foci-containing cells with >10 foci. The inset shows a representative staining of increased γ-H2AX foci (green) in siREV3L-transfected cells (bottom panels) compared to control siRNA-transfected cells (top panels). Cell nuclei were stained with DAPI (blue). Error bars indicate mean ± SEM (n = 3). *p < 0.05, **p < 0.01. (**B**) Under immunofluorescence microscopy, subcellular localization of BRCA1 was counted as having nuclear staining only, cytoplasmic staining only, or both nuclear/cytoplasmic staining. A total of 20 cells were counted per field and a total of 10 fields were assessed. Error bars indicate mean ± SEM (*n* = 3). *p < 0.05, siREV3L *vs*. combination of siREV3L and dacomitinib; **p < 0.01, siREV3L *vs*. combination of siREV3L and dacomitinib; ***p < 0.001, dacomitinib *vs*. combination of siREV3L and dacomitinib.

In addition to important roles in error-prone TLS in which DNA replication bypasses blocking lesions, REV3L has also been implicated in promoting repair of DNA DSBs by homologous recombination (HR)^24^,^25^. EGFR inhibitors have been reported to attenuate HR repair of DNA DSBs, resulting in persistent DNA damage^26^,^27^,^28^. Therefore, we investigated whether combination of siREV3L and dacomitinib can increase nuclear γH2AX foci, which is an *in situ* marker of DNA DSBs. In both cells, treatment with siREV3L alone or dacomitinib alone produced modest increase of γH2AX foci. Notably, cotreatment of siREV3L and dacomitinib significantly increased γH2AX foci, suggesting the significant reduction of HR-mediated DNA DSB repair (Fig. 6A).

BRCA1 is an essential component of HR repair of DNA DSBs and the nuclear function of BRCA1 is controlled by its subcellular localization^29^,^30^. To explore inhibition of HR repair, we observed BRCA1 cytoplasmic shuttling upon treatment of siREV3L alone, dacomitinib alone or their combination. In both cells, the inhibition of REV3L alone modestly increased cytoplasmic translocation of BRCA1. Remarkably, cotreatment of siREV3L and dacomitinib resulted in significant cytoplasmic translocation of BRCA1, preventing BRCA1-induced HR repair (Fig. 6B). Cytoplasmic retention of BRCA1 by cotreatment of siREV3L and dacomitinib was also confirmed by fluorescence microscopy (Supplementary Figure S6).

Taken together, these data suggest that the loss of REV3L function and dacomitinib treatment synergistically enhanced cytotoxicity via loss of HR repair of DNA DSBs.

## Conclusions

In this study, we performed WES in SCCHN patients treated with dacomitinib to identify potential predictive biomarkers. Extensive exome-wide studies in SCCHN have been reported^12^,^19^,^20^, uncovering recurrent somatic mutations in *TP53* (47–72%), *NOTCH1* (14–19%), *CDKN2A* (9–22%), *PIK3CA* (6–21%), *FBXW7* (5%), *HRAS* (4–8%) and *CASP8* (8%). However, most patient samples selected for sequencing are chemotherapy-naïve tumors, rendering the evaluation of chemotherapy effects on the cancer genomes difficult. Based on the hypothesis that cisplatin treatment may drive the mutational process and thereby promote tumor progression, we have profiled the complete genetic alterations in 18 cisplatin-pretreated SCCHN.

Using this approach, we described a ‘platinum’ mutational signature characterized by a predominance of C:G to A:T transversions in CCR sequence contexts. Several lines of evidence suggest that this unique signature is associated with cisplatin activity in the cancer genome. First, the ‘platinum’ signature is prominently observed in cisplatin-resistant genomes, but not in treatment-naïve SCCHN genomes. This signature is also unique when compared to the mutational signatures in more than 7,000 tumor samples from different cancer types^8^. Second, the sequence context for this signature fits remarkably with the known binding sites of cisplatin and the DNA repair process operative on cisplatin-DNA adducts. Third, the high mutation rate but low variant allele frequency is consistent with the mutational process associated with a potent mutagenic agent that is introduced to the cancer genomes at late stages of tumour progression.

Our study also suggests that REV3L may be a novel driver gene in SCCHN, the mutations of which are selected during cisplatin treatment and these mutations confer sensitivity to dacomitinib treatment. We offered the following evidences to support this conclusion. First, somatic mutation of *REV3L* is highly prevalent in our cohort of cisplatin-treated patients (3/18; 16.7%) compared to the treatment-naïve TCGA (8/279; 2.9%). This frequency is even higher when considering only the subset of cisplatin-resistant and dacomitinib-sensitive patients (3/7; 42.9%). Second, we independently confirmed all three *REV3L* mutations using Sanger sequencing in cisplatin-treated tumor samples but not in the cisplatin-naive samples from the same patients. This suggests that *REV3L* mutations or clonal selection is a consequence of cisplatin treatment. Third, we observed a significant increase in sensitivity to dacomitinib in two dacomitinib-resistant cell lines upon *REV3L* silencing. Thus, *REV3L* mutational status may be a promising predictive biomarker for dacomitinib in SCCHN.

*REV3L* is the gene encoding for the catalytic subunit of DNA polymerase zeta, an essential component in DNA translesion synthesis. DNA polymerase zeta has the remarkable abilities to efficiently bypass DNA lesions formed by cisplatin, UV, tobacco, AP site and others^31^,^32^. These properties allow cells with severely damaged DNA to survive, albeit at the cost of introducing large amount of mutations due to the error prone replication activity of DNA polymerase zeta. Therefore, somatic inactivation of REV3L may result in the accumulation of unrepairable DNA DSBs, which makes tumor cells vulnerable to dacomitinib-mediated inhibition of HR repair^21^,^27^,^28^. Indeed, our functional assays indicate that REV3L knockdown significantly increased the DNA DSBs, suggesting the loss of TLS. Administration of dacomitinib in REV3L-inactivated cells caused cytoplasmic retention of BRCA1, suggesting the additional loss of template-switching pathway.

Our study may be limited by small sample size. However, we believe that our study deserve special recognition due to following reasons. Firstly, all patients in our study had been enrolled and treated with dacomitinib in the prospective phase II trial^7^. Therefore, our study provides cleaner datasets and less selection bias. Secondly, all the tumor specimens were taken for exome sequencing immediately before the treatment of dacomitinib. Rebiopsy in heavily-pretreated cancer patients, despite its potential usefulness, poses significant clinical challenges due to patients’ reluctance to high-risk procedures, patient’s comorbidities and lack of biopsible tumor lesion^33^. Furthermore, genomic analysis of freshly rebiopsied tumors can be challenging due to limited amount of DNA extracted from the tumor tissues. These challenges explain why there have been few reports of comprehensive genomic study in metastatic cancer patients. Because of aforementioned reasons, there are a number of hurdles to confirm our findings in a large cohort.

Our results demonstrated a highly dynamic genetic landscape in heavily treated cancer cells. We showed that cisplatin treatment is likely responsible for the introduction of a massive amount of low frequency mutations with highly specific mutational signatures. We suggest a potential utility of this signature and provide a proof of principle that sequencing of previously treated tumor samples may uncover treatment-induced driver mutations as well as provide additional molecular targets for cancer therapy.

## Methods

### Patient Samples

Tumors and paired peripheral blood samples were collected prior to dacomitinib treatment in a prospective phase II trial (NCT01449201)^7^. All patients were Koreans. All patients signed consent forms for the sample collection and molecular analysis. The study was approved by the institutional review board of Severance Hospital, Seoul, Korea. All experiments were performed in accordance with relevant guidelines.

### Whole-exome sequencing and mutation analyses

Board-certified pathologists selected the cases with rich tumor cell populations to ensure the selection of regions >70% tumor purity. For genomic DNA extraction, we used the DNeasy Blood & Tissue Kit (Qiagen, Hilden, Germany) according to the manufacturer’s recommendation. For whole-exome sequencing, we used Agilent SureSelect Human All Exome 50Mb kit (Agilent Technologies). Using 200 ng of DNA, whole exome-sequencing was performed with Illumina HiSeq2000 platform to generate 150 bp paired-end sequencing reads according to the manufacturer’s instructions (Macrogen, Seoul, South Korea). To align the sequencing reads onto the reference genome (hg19), we used BWA (Burrows Wheeler aligner) based on Burrows-Wheeler transformation algorithm (v0.7.5a) using default options^34^. The local realignment of the sequencing reads were performed using Genome Analysis ToolKit (GATK; v3.1.-1)^35^ with known indel lists (Mills_and_1000G_gold_standard.indels.hg19.vcf and 1000G_phase1.indels.hg19.vcf) as available in GATK bundle datasets (ftp.broadinstitute.org We also used Picard v1.85 (http://picard.sourceforge.net) and SamTools v0.1.19 to sort the sequencing reads in order of genomic coordinates, to remove PCR duplicates and to fix the mate information of the paired-end sequence read. Score recalibration of the sequencing reads were also performed using GATK with known SNP sites (dbsnp_137.hg19.excluding_sites_after_129.vcf). In this study, we define mutations as somatic base substitutions (point mutations) and indels that are present in the tumor genome but absent in the matched normal genomes. By comparing the tumor sequencing reads with those of matched normal, we called somatic base substitutions using MuTect v1.1.4^36^. Somatic base substitutions were called using default log odds (LOD) score cutoff of 6.3 and 2.3 for the tumor and normal genomes, respectively, instead of predefined cutoffs of sequencing read depth. We used VarScan2 (v2.3.6) to call somatic indels also by comparing the sequencing reads of the tumor and matched normal genomes^37^. To obtain a list of confident indels, we selected indels whose allele frequencies are more than 0.2. ANNOVAR^38^ package was used to select the exonic mutations and also to predict their functional consequences such as silent or non-silent variants for somatic variants. Mutational rate was calculated assuming that the average exome has 30 Mb in protein coding genes with sufficient sequencing coverage. As control, we downloaded the somatic mutations of 279 HNSCC (lv2 somatic mutations) from the Cancer Genome Atlas consortium (https://tcga-data.nci.nih.gov/tcga/tcgaDownload.jsp).

### Deciphering the mutation signatures

For mutation signature analysis, we calculated the frequency of coding SNVs according to the 3 letter-based 96 trinucleotide classification system, i.e. the six mutation categories of base substitutions (C >A, C >G, C >T, T >A, T >C, and T >G) were further classified into 96 trinucleotides taking into consideration of 5′ and 3′ nucleotides immediate to the mutated base. The MatLab code for the mutation signature analysis was downloaded from a public resource (http://kr.mathworks.com/matlabcentral/fileexchange/38724-wtsi-mutational-signature-framework). To obtain the optimal number of mutation signatures, we performed permutation tests in which we measured the extent of signature stability in a set of 100 simulated cancer genomes by increasing the number of signature from one to ten (Additional file 5). Stable solution was observed up to five mutation signatures and we annotated five mutation signatures by comparing them with the previously reported mutation signatures identified from somatic mutations of cancer genomes^8^,^9^.

### The analysis of recurrent somatic mutations

The significantly mutated gene (smg) test in the Genome MuSiC (v0.4) suite^18^ was used using the default parameters to identify significantly mutated genes. Genes mutated in at least two patients and with a false discovery rate (FDR) adjusted convolution test *P* value of less than 0.2 were considered for further analysis.

### Reverse transcription PCR and real-time PCR assay

Expression of REV3L mRNA in the head and neck cancer cells was determined by real-time RT-PCR. Total RNA was extracted with RNeasy®Mini Kit (Qiagen) from the cell lines, and the initial cDNA strand synthesis was performed with SuperScript™ III First-Strand Synthesis SuperMix (Invitrogen) followed by kit manual. Real-time PCR was performed on Applied Biosystem Step One^TM^ with SYBR-Green PCR master mix (Life Technologies), using foward (5′-GCTCCAGTATGTGTACCATCTTGT) and reverse (5′-ATGGATATCTCGAAGTAACACGTC) primer. The expression levels of REV3L mRNA for each sample were determined by standardization with expression levels of GAPDH.

### Cell proliferation assay

Cellular sensitivity to dacomitinib was determined by the MTT cytotoxicity assay. Cells were plated in 96-well plates at 1 × 10^3^ cells/well and allowed to adhere for 24 h, then dacomitinib was added and incubated for 72 h. The medium was carefully removed and MTT solution was added to each well, and plates were incubated for 4 h. The medium was then replaced with DMSO, and the results were measured at 540 nm.

### Short interfering RNA transfection

siRNAs were synthesized to the target sequences of REV3L mRNA corresponding to REV3L siRNA #1 (5′-GAUCACAGGUUUGUGCCAG) and REV3L siRNA #2 (5′-AGACUGAGUGAGUCACCUG) (IDT). Cells were plated at a density of 0.5 × 10^6^ per 60 mm dish 1 day before transfection. siRNA was transfected into the cells using Lipofectamine RNAiMAX (Invitrogen) at a final concentration of 0.01 μM for 72 h, according to the manufacturer’s instructions.

### Cell cycle analysis

Cells were transfected with control siRNA or REV3L siRNA, and treated with or without dacomitinib for the indicated times. Following the treatment period, cell cycle distribution was determined using standard ethanol fixation and propidium iodide staining followed by flow cytometry.

### Clonogenic survival assay

Cells were transfected with either control siRNA or REV3L siRNA. Twenty four hours after transfection, cells were subjected to the treatment with either DMSO or the indicated dose of dacomitinib, paclitaxel or cisplatin for 14 days. Colonies were stained with crystal violet 0.005%.

### Immunofluorescence

Cells were transfected with control siRNA or REV3L siRNA, and treated with DMSO or 1 μM dacomitinib for 72 h. Following the treatment period, cell were fixed with 4% paraformaldehyde and permeabilized with ethanol. Primary antibodies include 200 μg/μl mouse anti-phospho-γ-H2AX antibody (Upstate), or 1:100 mouse anti-BRCA1 (Ab-1) (Calbiochem) after blocking with goat serum. Secondary antibodies include 1:1000 anti-mouse Alexa488 conjugated antibody (Molecular Probe) or 1:1000 anti-Rabbit Alexa594 conjugated antibody (Molecular Probe). Staining patterns were visualized via fluorescence microscopy (Olympus). A total of 20 cells were counted per field, and a total of 10 fields were assessed. For foci analysis, cells with >10 γH2AX foci were counted. For BRCA1 localization, cells were assessed as having nuclear staining only, cytoplasmic staining only, or both nuclear/cytoplasmic staining.

## Additional Information

**How to cite this article**: Huang, K. K. *et al.* Exome sequencing reveals recurrent *REV3L* mutations in cisplatin-resistant squamous cell carcinoma of head and neck. *Sci. Rep.* **6**, 19552; doi: 10.1038/srep19552 (2016).

## Supplementary Material

Supplementary files

Supplementary Table S4
