# Supplementary material for: Exome sequencing reveals recurrent REV3L mutations in cisplatin-resistant squamous cell carcinoma of head and neck
Source: Sci Rep. 2016 Jan 21;6:19552. doi: 10.1038/srep19552 (PMC4726344; doi:10.1038/srep19552)
Supplement: Supplementary files [file srep19552-s1.doc]

**Title**: Exome sequencing reveals recurrent REV3L mutations in cisplatin-resistant squamous cell carcinoma of head and neck

**Authors**:

Kie Kyon Huang^1,2^,*, Kang Won Jang^3^,*, Sangwoo Kim^4^, Han Sang Kim^5,6^, Sung-Moo Kim^3^, Hyeong Ju Kwon^7^, Hye Ryun Kim^7^, Hwan Jung Yun^8^, Myung Ju Ahn^9^, Keon Uk Park^10^, Kalpana Ramnarayanan^2^, John R. McPherson^2,11^, Shenli Zhang^2^, Je-Keun Rhee^12^, André L. Vettore^2^, Kakoli Das^2^, Takatsugu Ishimoto^2^, Joo Hang Kim^5^, Yoon Woo Koh^13^, Se Hun Kim^13^, Eun Chang Choi^13^, Bin Tean Teh^1,2,14^, Steven G. Rozen^2,11^, Tae-Min Kim^12^,**, Patrick Tan^1,2,15^** and Byoung Chul Cho^5^,**

*These authors contribute equally as first authors to this work.

**These authors contribute equally as corresponding authors to this work.

**Author affiliation:**

^1^Cancer Science Institute of Singapore, National University of Singapore, Center for Translational Medicine, Singapore, Singapore.

^2^Program in Cancer and Stem Cell Biology, Duke-NUS Graduate Medical School, Singapore, Singapore.

^3^JE-UK Institute for Cancer Research, JEUK Co., Ltd., Gumi-City, Kyungbuk, Korea

^4^Severance Biomedical Science Institute, Yonsei University College of Medicine, Seoul, Korea

^5^Division of Medical Oncology, Department of Internal Medicine, Yonsei Cancer Center, Seoul, Korea

^6^Department of Pharmacology, Pharmacogenomic Research Center for Membrane Transporters, Brain Korea 21 PLUS Project for Medical Science, Seoul, Korea

**^7^**Department of Pathology, Yonsei University College of Medicine, Seoul, Korea

^8^Department of Hematology-Oncology, Chungnam National University, Daejeon, Korea

^9^Division of Hematology-Oncology, Department of Medicine, Samsung Medical Center, Sungkyunkwan University School of Medicine, Seoul, Korea

^10^Department of Hematology-Oncology, Keimyung University, Daegu, Korea

^11^Duke-NUS Centre for Computational Biology, Duke-NUS Graduate Medical School, Singapore, Singapore.

^12^Department of Medical Informatics, College of Medicine, The Catholic University of Korea, Seoul, Korea

^13^Department of Otorhinolaryngology, Yonsei University College of Medicine, Seoul, Korea

^14^Laboratory of Cancer Epigenome, National Cancer Centre Singapore, Singapore, Singapore

^15^Genome Institute of Singapore, Singapore, Singapore

**Corresponding Author:**

Byoung Chul Cho, M.D., Ph.D. Yonsei Cancer Center, Division of Medical Oncology, Yonsei University College of Medicine, 50 Yonsei-ro, Seodaemun-gu, Seoul 120-752, Korea. E-mail: cbc1971@yuhs.ac

**Supplementary Table S1.** Clinicopathologic features (*n*= 18)

|  | |  |
| --- | --- | --- |
| Characteristic | No. of Patients | % |
| Sex |  |  |
| Male | 15 | 83.3 |
| Female | 3 | 16.7 |
| Age, years |  |  |
| Median (Range) | 61 | (40-78) |
| Performance status |  |  |
| 0 | 1 | 5.6 |
| 1 | 15 | 83.3 |
| 2 | 2 | 11.1 |
| Smoking history |  |  |
| Never smoker | 5 | 27.8 |
| Smoker, pack-years |  |  |
| ≤ 10 | 1 | 5.6 |
| > 10 | 12 | 66.6 |
| Primary site |  |  |
| Oral cavity | 8 | 44.4 |
| Oropharynx | 4 | 22.3 |
| Hypopharynx | 2 | 11.1 |
| Larynx | 2 | 11.1 |
| Maxillary sinus | 2 | 11.1 |
| Disease status at study entry |  |  |
| Locoregional | 9 | 50.0 |
| Distant | 1 | 5.6 |
| Both | 8 | 44.4 |
| Number of involved disease sites |  |  |
| 1 | 2 | 11.1 |
| 2 | 4 | 22.3 |
| ≥3 | 12 | 66.6 |
| Prior treatment |  |  |
| Cisplatin-based chemotherapy* | 18 | 100 |
| Radiation | 15 | 83.3 |
| Surgery | 17 | 94.4 |
| P16 status^§^ |  |  |
| Positive | 4 | 22.2 |
| Negative | 11 | 61.2 |
| Unknown | 3 | 16.6 |
| *Includes cisplatin alone, 5-fluorouracil *plus* cisplatin, docetaxel *plus* cisplatin, or docetaxel *plus* 5-fluorouracil *plus* cisplatin.  ^§^The p16 immunohistochemistry was used as a surrogate marker of HPV status of the tumor. | | |

**Supplementary Table S2.** Tumor response to dacomitinib (*n*= 18)

|  |  |  |
| --- | --- | --- |
| Characteristic | No. of Patients | % |
| Tumor response* |  |  |
| Complete response (CR) | 0 | 0 |
| Partial response (PR) | 1 | 5.6 |
| Stable disease (SD) | 13 | 72.2 |
| Progressive disease (PD) | 4 | 22.2 |
| Dacomitinib-sensitive** | 7 | 38.9 |
| Dacomitinib-resistant*** | 11 | 61.1 |
| *Response evaluations were defined according to RECIST 1.1 guidelines. Radiographic imaging was conducted at week 4, and every 8 weeks thereafter until disease progression or when clinically indicated.  **Defined as progression-free survival ≥4 months on dacomitinib.  ***Defined as progression-free survival <4 months on dacomitinib. | | |

**Supplementary Table S3.** Whole exome sequencing of 18 SCCHN tumor-normal pairs

| Samples* | Sequencing reads | Mapped (%) | Coverage (mean) | Coverage (median) | % of bases (>= 20 reads) |
| --- | --- | --- | --- | --- | --- |
| N1 | 58,136,147 | 52,222,949 (89.8%) | 82.43 | 71 | 86.8 |
| N2 | 61,638,697 | 54,090,004 (87.8%) | 90.14 | 78 | 87.8 |
| N3 | 35,776,907 | 33,494,969 (93.6%) | 54.57 | 48 | 80.9 |
| N4 | 60,126,362 | 54,141,351 (90.0%) | 90.46 | 79 | 88.4 |
| N5 | 66,750,808 | 58,837,587 (88.1%) | 95.74 | 83 | 88.4 |
| N6 | 60,153,486 | 54,415,825 (90.5%) | 90.29 | 78 | 88.2 |
| N7 | 81,185,116 | 72,698,625 (89.5%) | 119.39 | 104 | 90.6 |
| N8 | 59,589,874 | 53,664,251 (90.1%) | 89.21 | 77 | 88 |
| N10 | 53,774,256 | 49,946,027 (92.9%) | 83.56 | 73 | 87.6 |
| N11 | 74,899,117 | 68,005,810 (90.8%) | 112.45 | 96 | 90.3 |
| N12 | 51,692,410 | 43,795,608 (84.7%) | 75.78 | 65 | 85.6 |
| N13 | 56,006,449 | 51,357,745 (91.7%) | 85.19 | 74 | 88.2 |
| N14 | 60,775,860 | 57,162,696 (94.1%) | 91.1 | 80 | 89.5 |
| N15 | 64,309,256 | 57,844,960 (89.9%) | 96.44 | 84 | 89.4 |
| N16 | 64,927,110 | 58,131,883 (89.5%) | 97.85 | 86 | 89.2 |
| N17 | 68,985,289 | 61,685,006 (89.4%) | 103.54 | 89 | 89.5 |
| N18 | 64,259,454 | 56,963,267 (88.6%) | 97.04 | 84 | 88.8 |
| N19 | 61,814,056 | 56,588,065 (91.5%) | 94.23 | 82 | 88.9 |
| T1 | 97,089,528 | 87,729,952 (90.4%) | 137.95 | 106 | 87.3 |
| T2 | 108,949,162 | 98,650,220 (90.5%) | 158.85 | 112 | 86.7 |
| T3 | 100,100,217 | 90,298,687 (90.2%) | 141.93 | 104 | 86.2 |
| T4 | 100,056,774 | 89,587,545 (89.5%) | 141.8 | 111 | 88.5 |
| T5 | 98,483,362 | 88,227,376 (89.6%) | 141.7 | 99 | 84.9 |
| T6 | 95,588,228 | 86,289,694 (90.3%) | 136.95 | 93 | 83.1 |
| T7 | 88,764,390 | 79,878,969 (90.0%) | 126.18 | 100 | 87.9 |
| T8 | 94,548,871 | 91,568,160 (96.8%) | 137.86 | 104 | 87.7 |
| T10 | 88,154,184 | 77,397,508 (87.8%) | 124.51 | 97 | 87.5 |
| T11 | 92,564,857 | 83,563,694 (90.3%) | 132.33 | 99 | 85.8 |
| T12 | 94,141,848 | 83,951,845 (89.2%) | 135.33 | 100 | 85.4 |
| T13 | 100,778,013 | 91,395,099 (90.7%) | 144.84 | 103 | 85.4 |
| T14 | 137,977,452 | 121,462,420 (88.0%) | 179.5 | 128 | 86.9 |
| T15 | 145,467,750 | 129,221,651 (88.8%) | 189.32 | 136 | 88.5 |
| T16 | 95,770,782 | 83,733,010 (87.4%) | 137.77 | 104 | 86.1 |
| T17 | 132,684,221 | 116,600,601 (87.9%) | 169.78 | 130 | 89 |
| T18 | 193,447,204 | 172,434,148 (89.1%) | 251.56 | 179 | 90.3 |
| T19 | 103,514,851 | 96,780,683 (93.5%) | 157.68 | 136 | 92.9 |

| *The tumor and matched normal genomes are discriminated with the use of 'T' and 'N', respectively. |
| --- |
| **The mean and median coverage as well as the % of bases (>= 20 reads) were calculated onto the targeted regions (Agilent SureSelect 50Mb exon). |

**Supplementary Figure S5.** Genome MuSiC significance test in the overall cohort (*n*=18)

| #Gene | Indels | SNVs | P-value FCPT | P-value LRT | P-value CT | FDR FCPT | FDR LRT | FDR CT |
| --- | --- | --- | --- | --- | --- | --- | --- | --- |
| TP53 | 0 | 10 | 5.6E-16 | 0.0E+00 | 0.0E+00 | 1.0E-11 | 0.0E+00 | 0.0E+00 |
| TMPRSS13 | 3 | 2 | 2.8E-06 | 8.7E-10 | 2.9E-10 | 2.6E-02 | 3.9E-06 | 2.6E-06 |
| C10orf113 | 3 | 0 | 9.8E-05 | 3.0E-10 | 1.0E-08 | 4.5E-01 | 2.2E-06 | 4.7E-05 |
| GLYR1 | 3 | 0 | 1.8E-03 | 1.8E-08 | 1.2E-06 | 1.0E+00 | 6.7E-05 | 4.2E-03 |
| ELAVL3 | 2 | 1 | 5.0E-03 | 1.5E-06 | 2.7E-06 | 1.0E+00 | 3.9E-03 | 8.3E-03 |
| DPY19L3 | 0 | 3 | 8.4E-03 | 3.3E-06 | 8.5E-06 | 1.0E+00 | 6.5E-03 | 1.7E-02 |
| CDKN2A | 0 | 3 | 1.3E-02 | 7.0E-06 | 8.6E-06 | 1.0E+00 | 1.0E-02 | 1.7E-02 |
| RPS15 | 0 | 2 | 2.5E-02 | 1.2E-06 | 1.2E-05 | 1.0E+00 | 3.6E-03 | 2.2E-02 |
| ENTPD6 | 1 | 2 | 2.4E-02 | 1.4E-04 | 3.3E-05 | 1.0E+00 | 8.7E-02 | 5.2E-02 |
| TBP | 2 | 0 | 4.6E-02 | 3.6E-06 | 4.2E-05 | 1.0E+00 | 6.5E-03 | 5.5E-02 |
| FBXW7 | 0 | 3 | 2.6E-02 | 1.6E-04 | 4.2E-05 | 1.0E+00 | 9.1E-02 | 5.5E-02 |
| HIST1H3H | 0 | 2 | 5.6E-02 | 6.4E-05 | 6.3E-05 | 1.0E+00 | 5.3E-02 | 7.6E-02 |
| ARRDC5 | 1 | 1 | 6.1E-02 | 7.3E-05 | 8.5E-05 | 1.0E+00 | 5.7E-02 | 9.1E-02 |
| MAMSTR | 2 | 0 | 5.6E-02 | 5.1E-06 | 9.4E-05 | 1.0E+00 | 8.4E-03 | 9.5E-02 |
| NOP58 | 2 | 0 | 7.5E-02 | 9.1E-06 | 1.2E-04 | 1.0E+00 | 1.1E-02 | 1.1E-01 |
| TM4SF18 | 0 | 2 | 8.3E-02 | 1.3E-04 | 1.2E-04 | 1.0E+00 | 8.7E-02 | 1.1E-01 |
| IRF5 | 2 | 0 | 7.2E-02 | 8.1E-06 | 1.6E-04 | 1.0E+00 | 1.0E-02 | 1.4E-01 |
| KRAS | 0 | 2 | 9.8E-02 | 1.6E-05 | 1.8E-04 | 1.0E+00 | 1.7E-02 | 1.4E-01 |
| CRIPAK | 0 | 2 | 1.1E-01 | 2.1E-04 | 1.7E-04 | 1.0E+00 | 1.0E-01 | 1.4E-01 |
| ZNF658 | 0 | 2 | 9.0E-02 | 1.3E-05 | 2.1E-04 | 1.0E+00 | 1.5E-02 | 1.5E-01 |
| CNTN6 | 0 | 3 | 7.5E-02 | 1.4E-04 | 2.2E-04 | 1.0E+00 | 8.7E-02 | 1.5E-01 |
| NR1H4 | 0 | 2 | 1.1E-01 | 2.1E-04 | 2.4E-04 | 1.0E+00 | 1.0E-01 | 1.5E-01 |
| DNMT1 | 2 | 1 | 6.2E-02 | 8.3E-05 | 2.7E-04 | 1.0E+00 | 6.3E-02 | 1.6E-01 |
| CDK14 | 0 | 2 | 1.1E-01 | 2.2E-04 | 2.8E-04 | 1.0E+00 | 1.0E-01 | 1.6E-01 |

**Supplementary Figure S6.** Genome MuSiC significance test in the dacomitinib-responsive cohort (*n*=7)

| #Gene | Indels | SNVs | P-value FCPT | P-value LRT | P-value CT | FDR FCPT | FDR LRT | FDR CT |
| --- | --- | --- | --- | --- | --- | --- | --- | --- |
| TP53 | 0 | 4 | 2.9E-05 | 1.1E-07 | 3.2E-09 | 5.2E-01 | 2.1E-03 | 5.9E-05 |
| TBP | 2 | 0 | 1.4E-02 | 4.6E-07 | 4.3E-06 | 1.0E+00 | 4.1E-03 | 3.9E-02 |
| EPYC | 0 | 2 | 4.5E-02 | 3.6E-06 | 5.0E-05 | 1.0E+00 | 1.7E-02 | 1.2E-01 |
| REV3L | 2 | 1 | 2.5E-02 | 1.9E-05 | 3.8E-05 | 1.0E+00 | 5.2E-02 | 1.2E-01 |
| CDKN2A | 0 | 2 | 6.7E-02 | 8.9E-05 | 5.4E-05 | 1.0E+00 | 1.5E-01 | 1.2E-01 |
| HIST1H3H | 0 | 1 | 2.6E-01 | 1.1E-04 | 4.3E-05 | 1.0E+00 | 1.6E-01 | 1.2E-01 |

**Supplementary Figure S7.** Genome MuSiC significance test dacomitinib-resistant cohort (*n*=11)

| #Gene | Indels | SNVs | P-value FCPT | P-value LRT | P-value CT | FDR FCPT | FDR LRT | FDR CT |
| --- | --- | --- | --- | --- | --- | --- | --- | --- |
| TP53 | 0 | 6 | 6.0E+00 | 1.3E+04 | 4.5E+02 | 5.2E-09 | 5.6E-13 | 6.6E-14 |
| C10orf113 | 3 | 0 | 3.0E+00 | 4.9E+03 | 6.2E+02 | 3.6E-05 | 7.5E-11 | 3.0E-09 |
| TMPRSS13 | 2 | 2 | 4.0E+00 | 1.9E+04 | 2.1E+02 | 1.1E-04 | 8.0E-08 | 3.2E-08 |
| ARRDC5 | 1 | 1 | 2.0E+00 | 1.1E+04 | 1.8E+02 | 3.9E-02 | 3.3E-05 | 4.2E-05 |
| IRF5 | 2 | 0 | 2.0E+00 | 1.7E+04 | 1.2E+02 | 4.4E-02 | 3.2E-06 | 5.4E-05 |
| TM4SF18 | 0 | 2 | 2.0E+00 | 6.8E+03 | 2.9E+02 | 5.8E-02 | 6.8E-05 | 5.9E-05 |
| GLYR1 | 2 | 0 | 2.0E+00 | 1.9E+04 | 1.1E+02 | 4.9E-02 | 3.9E-06 | 7.2E-05 |
| CNTN6 | 0 | 3 | 3.0E+00 | 3.5E+04 | 8.6E+01 | 4.9E-02 | 6.4E-05 | 8.2E-05 |

**Supplementary Figure S1. The results of stability test and the contribution of the five predicted mutational signatures to individuals SCCHN genome**


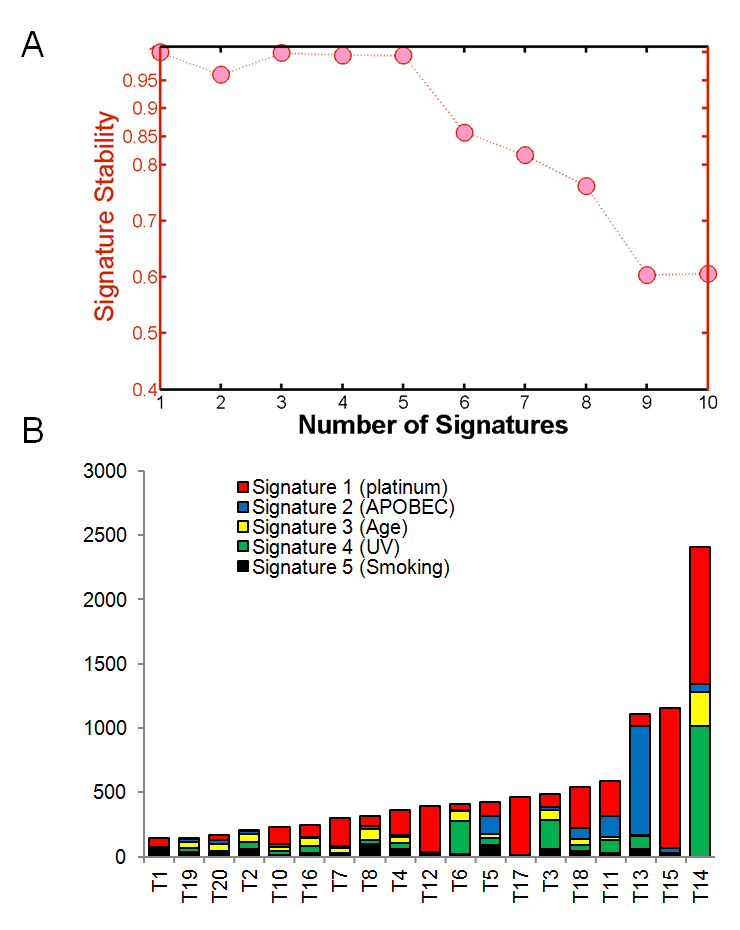


(A) The optimal number of mutational signatures can be determined by permutation tests. The plot shows that increasing the number of mutation signatures to five does not substantially reduce the signature stability suggesting that five deciphered signatures are reproducible.

(B) Each bar represents a SCCHN tumor sample. Vertical axis denotes the number of mutations per sample contributed by distinct mutational processes as indicated in the legend.

**Supplementary Figure S2. Sanger validation of REV3L mutations in pre- and post-cisplatin treated tumors**


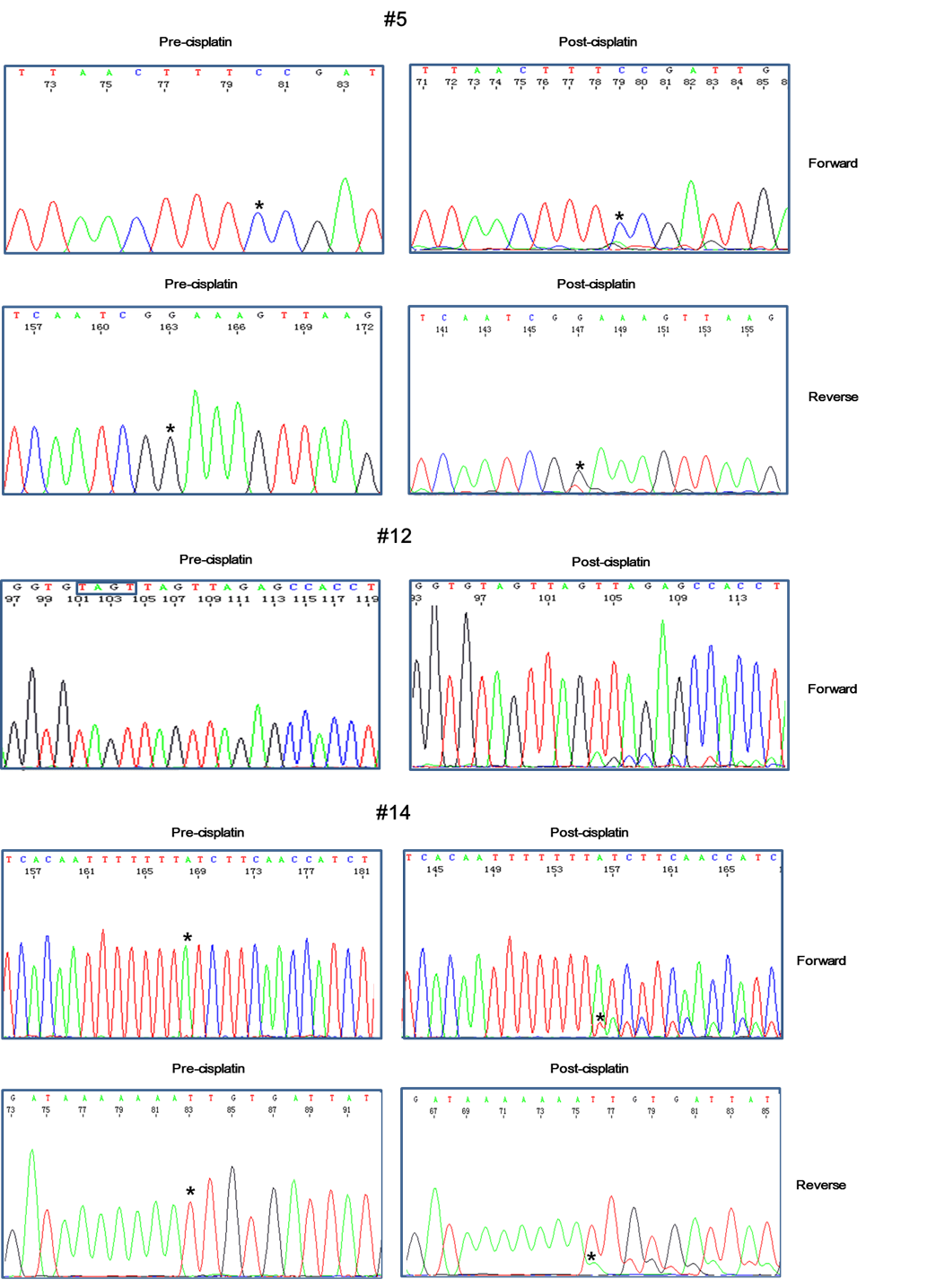


**Supplementary Figure S3. mRNA expression of REV3L and cell viability to dacomitinib in head and neck cancer cell lines.**


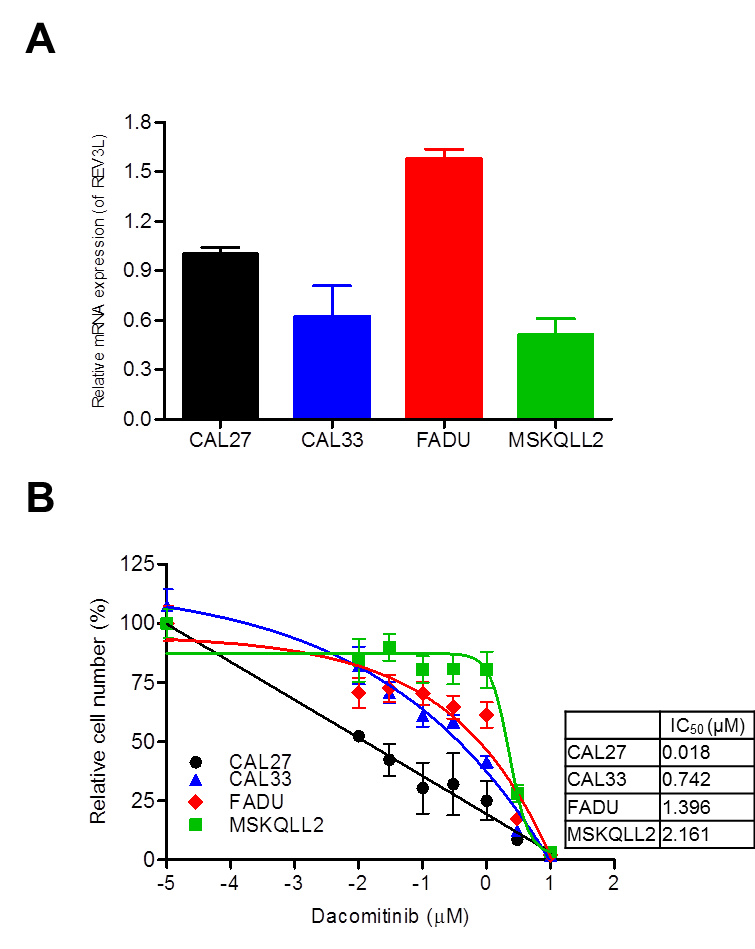


**(A)** Relative expression of REV3L mRNA in head and neck cancer cell lines (CAL27, CAL33, FADU, and MSKQLL2). Expression of REV3L mRNA was determined by real-time RT-PCR analysis. Each data is the mean of three independent experiments.

**(B)** Growth inhibition of head and neck cancer cell lines after 72 h treatment with dacomitinib at the indicated concentration. Values are relative to dimethyl sulfoxide (DMSO) controls and IC_50_ values are mean (μM; *n* = 3) ± SEM.

**Supplementary Figure S4. Combination of siREV3L and dacomitinib induces senescence in FADU and MSKQLL2 cells.**

**
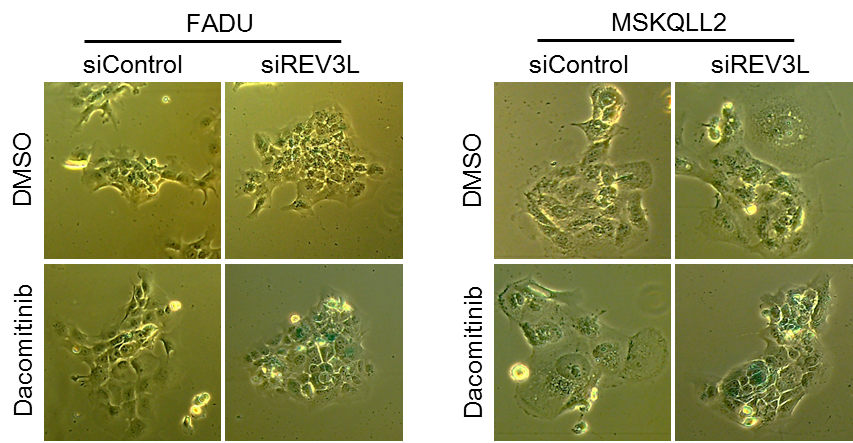
**

After 24 hours of transfection with control siRNA and siREV3L, cells were treated with dacomitinib for 72 h. Then, cells were stained for β-gal and micro-photographed.

**Supplementary Figure S5. REV3L depletion did not enhance the growth inhibitory effects of paclitaxel or cisplatin in head and neck cancer cells.**


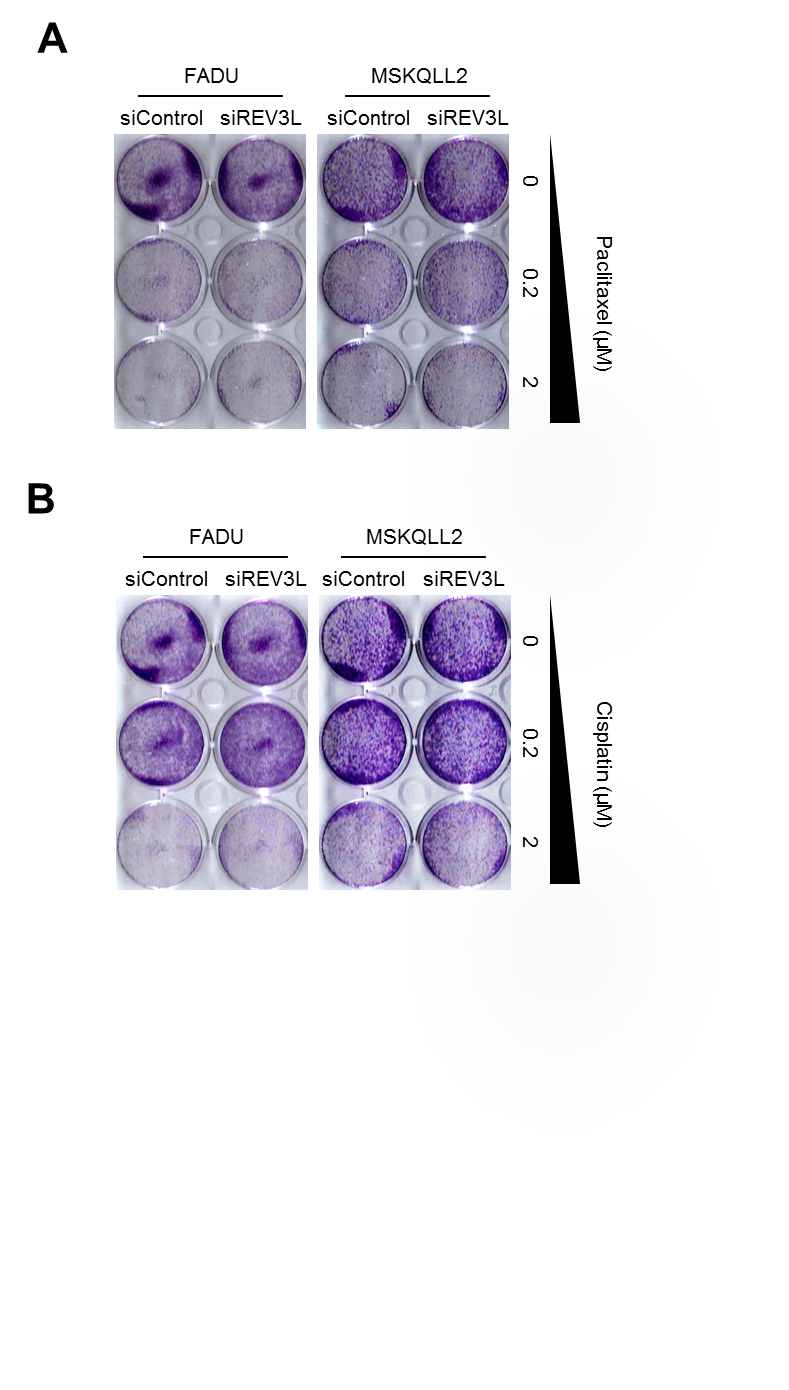


**(A)** Cells were transfected for 24 h with control siRNA or siREV3L, respectively, and were treated with or without paclitaxel at the indicated concentration for 14 days. Following the treatment, cells were fixed and stained with crystal violet 0.005%.

**(B)** Cells were treated with or without cisplatin at the indicated concentration for 14 days after transfection with control siRNA or siREV3L, respectively. Following the treatment, cells were fixed and stained with crystal violet 0.005%.

**Supplementary Figure S6. Dacomitinib translocates BRCA1 from nucleus to cytoplasm in REV3L- silenced head and neck cancer cells.**

**
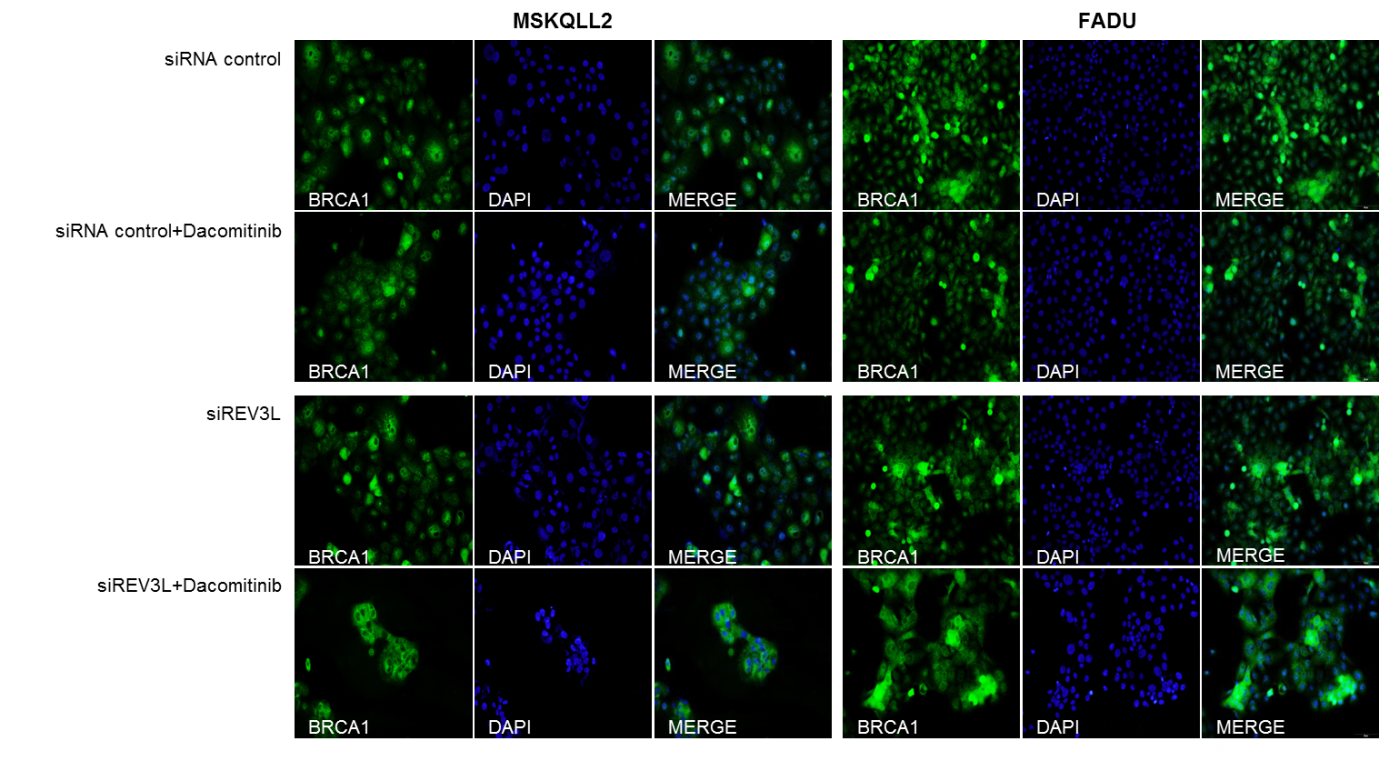
**

Cells were transfected with control siRNA or siREV3L for 24h, and were treated with or without dacomitinib 1 μM for 72 h. Following the treatment, cells were stained with BRCA1 antibody (green) and DAPI (blue), and the subcellular localization of BRCA1 was detected by fluorescence microscopy.
